# From assessment to improvement of elderly care in general practice using decision support to increase adherence to ACOVE quality indicators: study protocol for randomized control trial

**DOI:** 10.1186/1745-6215-15-81

**Published:** 2014-03-19

**Authors:** Saeid Eslami, Marjan Askari, Stephanie Medlock, Derk L Arts, Jeremy C Wyatt, Henk CPM van Weert, Sophia E de Rooij, Ameen Abu-Hanna

**Affiliations:** 1 Department of Medical Informatics, Academic Medical Center, University of Amsterdam, Amsterdam, The Netherlands; 2 Pharmaceutical Research Center, School of Pharmacy, Mashhad University of Medical Sciences, Mashhad, Iran; 3 Department of General Practice, Academic Medical Center, University of Amsterdam, Amsterdam, The Netherlands; 4 Leeds Institute of Health Sciences, University of Leeds, Leeds, UK; 5 Department of Geriatrics, Academic Medical Center, University of Amsterdam, Amsterdam, The Netherlands

## Abstract

**Background:**

Previous efforts such as Assessing Care of Vulnerable Elders (ACOVE) provide quality indicators for assessing the care of elderly patients, but thus far little has been done to leverage this knowledge to improve care for these patients. We describe a clinical decision support system to improve general practitioner (GP) adherence to ACOVE quality indicators and a protocol for investigating impact on GPs’ adherence to the rules.

**Design:**

We propose two randomized controlled trials among a group of Dutch GP teams on adherence to ACOVE quality indicators. In both trials a clinical decision support system provides un-intrusive feedback appearing as a color-coded, dynamically updated, list of items needing attention. The first trial pertains to real-time automatically verifiable rules. The second trial concerns non-automatically verifiable rules (adherence cannot be established by the clinical decision support system itself, but the GPs report whether they will adhere to the rules). In both trials we will randomize teams of GPs caring for the same patients into two groups, A and B. For the automatically verifiable rules, group A GPs receive support only for a specific inter-related subset of rules, and group B GPs receive support only for the remainder of the rules. For non-automatically verifiable rules, group A GPs receive feedback framed as actions with positive consequences, and group B GPs receive feedback framed as inaction with negative consequences. GPs indicate whether they adhere to non-automatically verifiable rules. In both trials, the main outcome measure is mean adherence, automatically derived or self-reported, to the rules.

**Discussion:**

We relied on active end-user involvement in selecting the rules to support, and on a model for providing feedback displayed as color-coded real-time messages concerning the patient visiting the GP at that time, without interrupting the GP’s workflow with pop-ups. While these aspects are believed to increase clinical decision support system acceptance and its impact on adherence to the selected clinical rules, systems with these properties have not yet been evaluated.

**Trial registration:**

Controlled Trials NTR3566

## Background

### Quality of care in elderly patients

Within the present fragmented healthcare organization, elderly patients and especially vulnerable elderly patients with complex problems often receive suboptimal care coordination and management. This may lead to premature functional decline or deterioration of quality of life [1]. Elderly people are in need of an effective, continuous, integrated and comprehensive package of preventive and curative health care. This requires constant efforts to assess and improve the quality of their care. The study, Assessing care of vulnerable elders (ACOVE) has process-based quality indicators (QI) that comprise a promising system for the comprehensive assessment of quality of care. The current version of the indicators, ACOVE-3, includes 392 indicators. These indicators are meant to capture minimal standards of care - standards that, if not met, almost ensure that the care is of poor quality [2]. These indicators were developed and judged by clinical experts to improve patient outcomes on the basis of clinical evidence and professional opinion [2]. The ACOVE QIs are in essence a list of *if-then* statements, linking a logical condition to a conclusion. The indicator is adhered to if the conclusion (which is usually an action) is fulfilled when the indicator is eligible (that is, when its logical condition is true). Some QIs are accompanied by an explanation; an example of such a QI is: ‘*If* a vulnerable elder is admitted to a hospital or is new to a physician practice, *then* multidimensional assessment of cognitive ability and assessment of functional status should be documented *because* screening for dementia can lead to early detection and initiation of treatment that may delay further progression.

ACOVE is emerging as an international standard for quality of care assessment for elderly patients. There are increasing numbers of studies that have translated the QIs to various settings [3]. In the Netherlands, the Trimbos Institute initiated selection and translation of rules relevant for the care of vulnerable elders in Dutch general practice, resulting in 81 QIs covering eight domains (dementia, depression, osteoporosis, osteoarthritis, medication management and use, hearing loss, continuity of care and falls) [4].

### From assessment to improvement

QIs are predominantly used for assessment of quality of care relatively long after it has been delivered, rather than directly improving care [3]. It remains unclear how and to what extent such quality assessment can contribute to actually improving the quality of care. In our project we want to exploit QIs to proactively influence physicians’ behavior at the time of care provision. To accomplish this, we will represent QIs in computer interpretable clinical rules (CRs) and incorporate them in a real-time clinical decision support system (CDSS), integrated in care providers’ information system. By matching patient data and physicians’ behavior to the clinical rules, the CDSS can provide feedback to the physicians in the form of alerts and reminders. This feedback is meant to help general practitioners (GPs) adhere to the clinical rules, and hence to the underlying QIs. Implementation of a CDSS at the point of care is likely to improve adherence to the clinical rules and thereby improve the overall quality of care. Currently, however, there are no computerized decision-support approaches to proactively support physicians for improving care for older patients on a comprehensive set of QIs aimed at preventing functional decline.

### Decision-making tools and appropriate practice/optimal care delivery

The potential effectiveness of CDSSs has been demonstrated by various studies [5]. In a series of studies we have also demonstrated the feasibility and effectiveness of computer-generated feedback in the Intensive Care Unit on the quality of care provided [6-8]. However, computer-generated feedback that is too often irrelevant or intrusive may result in alert fatigue and irritate clinicians. Alert fatigue is defined as the mental state that results from too many alerts and expenditure of mental energy, which can cause relevant alerts to be unjustifiably overridden along with clinically unimportant ones [9]. We relied on active end-user involvement in selecting the rules to support and on a model for providing feedback displayed as color-coded real-time messages concerning the patient visiting the GP at that time, without interrupting the GP’s workflow with pop-ups. While these aspects are believed to increase CDSS acceptance and its impact on adherence to the selected CRs, systems with these properties have not yet been evaluated.

### The primary care centers GAZO

In the Netherlands all inhabitants are enlisted with a GP, who is responsible for comprehensive health care and acts as a gatekeeper for secondary care. *Gezondheidcentra Amsterdam Zuid-Oost* (GAZO) comprises six primary care health centers in the southeast region of Amsterdam. A total of 45 GPs work in the GAZO centers (full and part-time). Every patient belongs to only one GP. However, when a GP is absent, another GP from a designated team of GPs may care for his or her patients. The team consists of pairs or triplets of GPs sharing the care of the same patients. There is an average of 7,450 (range: 3,285 to 10,055) registered patients in each center. About 10% of the patients are 65 years or older and about 5% are 75 years or older (data originating from 2009).

### Study aims

The primary aim of this study is the systematic improvement of the quality of primary care for older persons (defined as 65 years of age or older) by increasing adherence to the ACOVE-based CRs. To attain this increase in adherence we intend to use a CDSS that uses the respective CRs in combination with patient and treatment data to proactively support health care professionals to make the right decisions at the right time.

## Methods

### Preparation

#### Selecting the relevant CRs

We used a modified Delphi method to select the most relevant QIs from the original set of 81 QIs that were translated to Dutch general practice [4]. Table 1 (Prioritization) shows the steps of this part of the project. To assess the perceived need for a QI, we asked GPs whether they would personally switch decision support for the corresponding rule *on* or *off*. This is a more direct approach to understand their intention to comply than asking about importance and relevance of the rules.

**Table 1:** Ordered tasks and activities in this study

**Tasks**	**Activities**
**Prioritization**	1. Distribute brief questionnaires among 10 general practitioners (GPs) to select relevant quality indicators (QIs) out of 81 QIs
	2. Discuss result of phase 1 and disagreements in a focus group
	3. Distribute detailed questionnaires among end-users to select clinical rules (CRs) from those in phase 1
**Design and implementation of clinical decision support system (CDSS)**	4. Design triggering points in the GP’s electronic medical record
	5. Design CDSS for selected CRs in CR engine
	6. Implement and test CDSS in elecronic medical record
**Measuring baseline**	7. Start gathering the data for selected verifiable CRs and measure baseline before activating the CDSS
**Starting the trial**	8. Randomize and pilot CDSS with two users and gather reasons for non adherence
	9. Tune the CDSS based on phase 8 and start randomized controlled trial

#### Implementability of relevant QIs in computer-based CRs

We recently introduced the logical elements rule method (LERM) as a step-by-step method for assessing the amenability of CRs for decision support use, and to formalize the rules in a computer-based form [10]. We use LERM in this project to identify the implementable QIs and translate them to CRs in a form usable for a computer (hereafter referred to simply as CR).

#### Design and implementation of the CDSS

Table 1 (Design and implementation of CDSS) shows the different steps of this second part of the project. We have developed a software module, which is not an integral part of the GPs’ electronic medical records (EMR). It forms a plug-in that interfaces with a specific EMR type. We use the same plug-in in our other trial for improving stroke prevention in patients with atrial fibrillation [11] and shortly describe it here. The CDSS plug-in can be classified [12] as active (it provides feedback without being asked to), and non interruptive (it does not interrupt the workflow of the GP). For most rules it operates in the critiquing mode (its feedback is based on deviations from the QIs) but for some rules it works in the consulting mode by providing timely advice before a decision has been made. The GPs’ EMR system includes trigger points reacting on events such as submitting a medication prescription. These triggers are: opening a patient's electronic medical record on the computer, the addition of laboratory data, changing medication, and updating the patient’s problem list. Such events will trigger sending relevant data to a remote server, called the clinical rule engine (CRE) via a secure Internet connection. This information consists of: the data buffer that holds the information entered or selected by the GP that is shown on the screen at that moment (such as selection of a medication) but is not yet saved permanently to the EMR database, laboratory data from the last 12 months, the active medication list, and a list of the patient’s diagnoses codes. The CRE evaluates the information originating from the EMR by the computerized decision rules and sends, in response, a message in an eXtensible markup language (XML) file to the CDSS plug-in on the GP’s computer. The plug-in extracts the message title from the XML file, and displays a shortened form of the title in a thin sidebar that is attached to the right side of the GP’s screen. We refer to this sidebar as the dynamic floating list (DFL) because it is updated in real-time and can be moved around on the screen. The GP can move the mouse cursor over the DFL to display the full message title and can click on it in order to open the whole message in another window. The message usually includes advice and relevant findings supporting the advice. In addition, if the GP wishes to overrule the advice, he or she also has the possibility to indicate on this window why they do not wish to adhere to the rule. Upon first appearance, the message title is displayed with a red background in the DFL, indicating that the message is new. If the GP does not open the message or does not behave according to its advice, the background will become orange the next time the GP opens the EMR, indicating the message has been shown before but not yet dealt with. For an automatically verifiable real-time rule, when the GP changes the treatment plan according to the guideline, the background will turn green and the message will then disappear. This can happen without opening the message, for example the GP might already know that he or she needs to change the treatment plan and doing so will also result in the message disappearing, without having to interact with the CDSS plug-in.

### End-user involvement

Social aspects in which computerized applications operate are thought to be influential on the degree of success of these applications [13]. In particular, users’ preferences and concerns should be taken into account in the design and implementation of such applications. This contributes to reducing their resistance to the change introduced by the system. In our project, end users (GPs) are invited to help select the CRs for which they perceive a genuine need for decision support, using questionnaires and focus groups. In addition, we will analyze which factors make CRs more likely to be selected by GPs for decision support (such as perceived external social pressure to do well on a particular CR, high probability of forgetting the action required by a CR, et cetera).

### Baseline measurements

Baseline adherence to automatically verifiable CRs will be based on data elements residing in the central EMR database of the primary care centers in GAZO (EMRs of the GAZO centers are maintained by an external central server). We will measure baseline adherence based on at least 24 months prior to the use of the CDSS.

### Experiments

**Figure 1:**
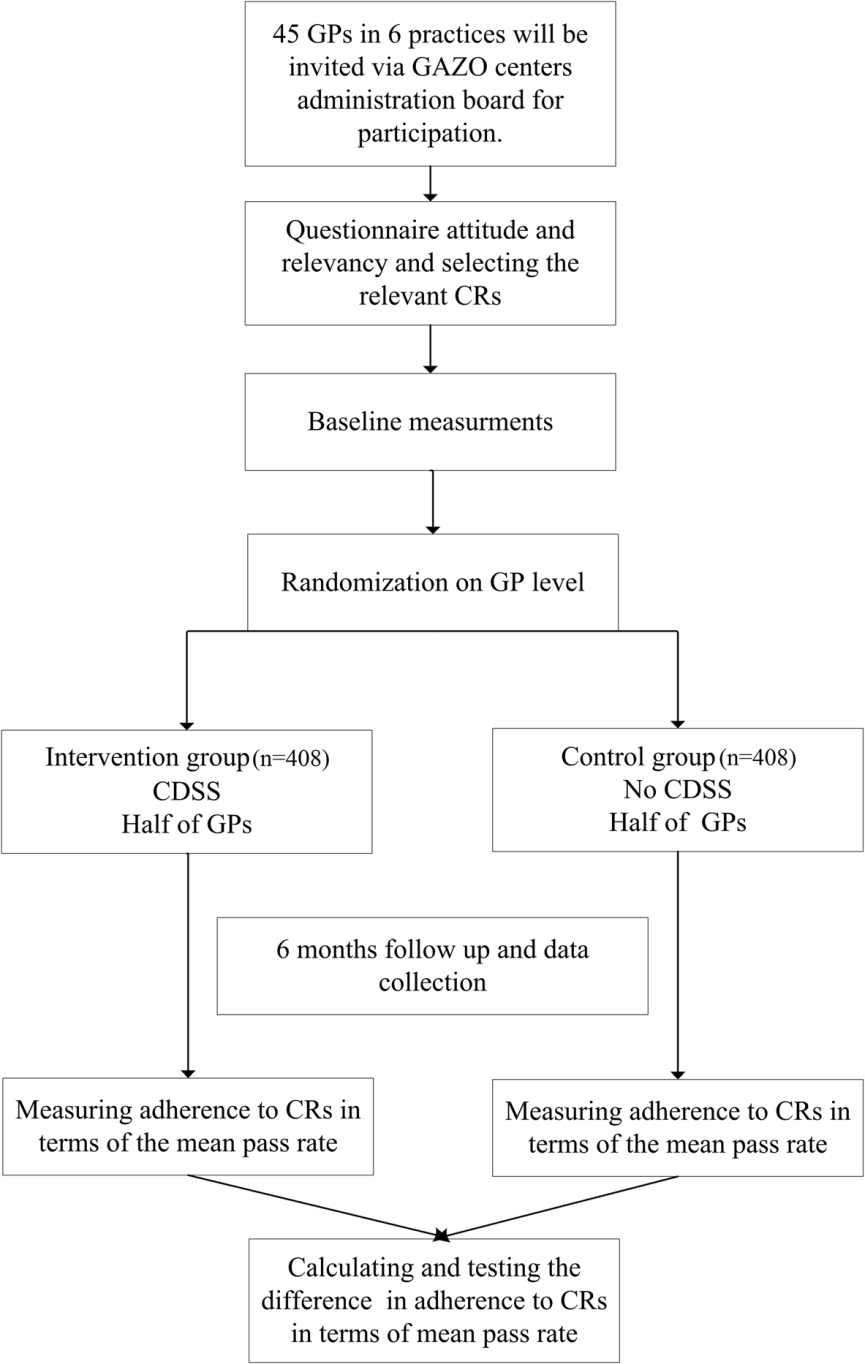
Consort diagram of the trial.

This prospective interventional study has a two-outcome assessor-blinded randomized controlled trial design (Figure 1). The first trial pertains to verifiable CRs. Verifiable CRs are ones for which the CRE is able to verify in real time whether the GP adheres to them or not. For example, the rule: “*If* an elder is treated with a non-steroidal anti-inflammatory drug, *then* s/he should be treated concomitantly with either misoprostol or a proton pump inhibitor” is verifiable because the CRE can establish whether one of the indicated medications was prescribed. In contrast, consider the CR, “*All* (vulnerable) elders should have an annual drug regimen review.” This CR is unverifiable by the CRE because medication review is not recorded in a structured way in the EMR. The second trial pertains to non-automatically verifiable CRs.

For a verifiable CR that is not adhered to, the CRE will provide a feedback intervention to the GPs for which the CR is active (that is, the GPs are in the intervention group for that CR). The feedback implies updating the items and/or colors of the DFL.

For an unverifiable CR, the only way to establish whether the GP adheres to it is to ask the GP whether he or she adheres to the rule. However, because we cannot establish the extent of adherence to these CRs at baseline, the CRE will randomize feedback provision using two types of framing for the advice: framing advice as action with positive (AP) consequences versus framing it as inaction with negative (IN) consequences. For example, for the annual drug regimen review CR described above, the AP framing will be: “Documenting weight loss can allow earlier detection of malignancy and malnutrition” and the IN framing will be “Failure to document weight loss can lead to delayed detection of malignancy and malnutrition.” In this way we will still be able to compare the (self-reported) adherence to the rules in both types of framing.

#### Bias control and randomization

Randomization will take place after measurement of baseline data. Every CR will be randomized into two groups of GP teams. Recall that while a patient will usually be seen by only one GP, a GP team consists of two or three GPs sharing care for the same sub-population of patients. When a patient’s official GP is absent, another GP from the team may see the patient.

Based on the baseline measurement and the expected room for improvement, randomization at the GP level is the most appropriate choice in terms of power and also practicality. However, this randomization choice will be adapted in order to control for possible contamination and biases. The discussion below pertains to verifiable CRs because they are the most prone to contamination.

We assume that contamination between GPs, especially in the same center, is relatively high when some GPs receive support for a rule (intervention) and the others none (control). To reduce the probability of this kind of contamination we have two groups of GPs, A and B. Every GP in group A will receive support for a specific subset of supported CRs (subset A) and no support for the rest of the CRs (subset B). By the same token, Every GP in group B will receive support for subset B and no support for subset A. This means that for about half the CRs (subset A) the GPs in group A form the intervention group and the GPs in group B form the control group, and vice versa for the CRs in subset B. All GPs will hence receive some kind of support, which means they do not have to feel that they are in the control group. A disadvantage of this approach is that some CRs are inter-related and improvement in one can explicitly or implicitly lead to improvement in the related one, even if it is not supported. For example, if one CR is supported by giving a reminder to order a laboratory test, and the same laboratory test is required by another CR that is not supported, ordering the laboratory test will be registered as adherence to both CRs. Therefore we will group all inter-related subsets of CRs in either subset A or subset B, elements of the inter-related subsets will hence never appear in both groups.

Finally, although patients usually only see their own GP, the GPs do work in teams. If a patient of one of these GPs requires an immediate appointment, they may see another *paired* GP. To avoid this potential contamination, we will ensure that GPs in a team will be randomized to the same subset. As for the non verifiable CRs, group-A GPs will receive the AP (action-positive) framing, and group-B GPs will receive the IN (inaction-negative) framing. Randomization of GP teams will be concealed; a person who is not responsible for recruiting subjects and has no knowledge of the study conduct will perform it on a computer.

### Participants

#### Patients

This study will include all patients who are 65 years and older in six GP centers. Note that although the ACOVE QIs were meant for vulnerable elders, most of these rules (and certainly the ones that will be selected) will be relevant to all elderly patients, vulnerable or not.

#### Physicians

All GPs in the GAZO centers will be included in the study. All GPs use the same GP information system in their practice.

### Outcome measure

The main outcome measure is the degree of adherence to the automatically verifiable clinical rules (regardless of placement of GPs in teams or centers). Adherence will be calculated in the following ways: 1) as the first primary outcome measure, on which the trial will be powered, we will calculate adherence in terms of the mean pass rate, without distinguishing between specific rules. For this outcome we simply divide the number of times any rule was followed by the number of times any rule was eligible to be followed; 2) we will calculate the pass rates for each rule separately as the second primary outcome measure. The pass rate of a rule is the proportion of times that a rule was followed when it was eligible; and 3) as a secondary outcome we will calculate the number of rules for which there was effect (the pass rate of a rule in its corresponding intervention group was different than in its control group).

We will also calculate the abovementioned measures per patient. Measures pertaining to fall prevention and management will also be investigated separately because fall management comprises a distinct project in itself.

The main outcome measure for the non-verifiable CRs is the degree of adherence as reported by the GPs themselves. In particular, upon receiving advice the GPs can indicate whether they agree (and hence adhere) or disagree (and hence not adhere) with the advice. Adherence will be calculated in the following ways: 1) adherence in terms of the mean pass rate as the first primary outcome measure; 2) adherence for each rule separately, as the second primary outcome; and 3) the number of rules for which there was effect, as a secondary outcome.

### Determination of sample size

The determination of sample size is dependent on the definition of the statistical unit of interest, the estimated incidence of triggers in the study population and a hypothesis about the effectiveness of the intervention.

#### Statistical unit

The main aim of this study is testing the change in adherence to the automatically verifiable CRs compared to the adherence at baseline of GPs, in terms of the mean pass rate of the rules, and the pass rate per rule. These CRs belong to different domains of geriatric care. Therefore, it is meaningful to consider a relevant subset of the selected verifiable CRs as the main statistical unit in order to calculate the sample size. This means that we eventually aim at increasing the average of the adherence to these CRs.

#### Hypothesis about the efficacy of the intervention

A *case* is defined as the situation in which a CR is eligible, meaning that its logical condition is true. Numerous CRs from the same patient can be assessed, and eligibility cases of the same CR are considered to be mutually independent. A power calculation was performed to determine the number of cases needed to detect a minimally clinically relevant effect of the CDSS based on the previously published studies. So far, no studies have been performed that measure the level of adherence to a set of ACOVE QIs in primary care in The Netherlands but Wenger *et al*. [14] showed that a practice-based intervention in the primary-care setting had mean absolute increase pass rates of 21% (from 23 to 44%) and 15% (from 22 to 37%) in two sets of ACOVE CRs.

We assumed 40% adherence in the control group (based on our preliminary baseline analysis) in both verifiable and non verifiable CRs. We can hence make a conservative estimate of an absolute increase of 10% in the mean pass rate of the supported set of CRs in the intervention group compared to the control group. For non-cluster randomization, for a power of 0.80, and two-sided testing at the 0.05 significance level, a total of 408 cases in which CRs are eligible are required in each group.

### Statistical analyses

Because the included CRs in each trial are not comparable, we do not compare the result of the verifiable CRs trial with the non-verifiable CRs trial. During the analysis plan we therefore did not consider the dependency between the hypotheses behind the two types of trials. The chi-square test will be used to compare individual and sets of CR pass rates between the supported and non supported automatically verifiable CRs, and between the CRs with advice that was framed differently (AP and IN). The binomial test will be used to test whether the number of supported CRs had higher pass rates than the non supported CRs. Statistical analyses will be performed with the R statistical software (R Foundation for Statistical Computing, Vienna, Austria). Standard statistical tests will be used to compare the baseline characteristics of the centers and patients.

### Time line

Table 1 shows the sequence of steps in the project. It started in September 2013, will need at least 6 months of trial, and we plan to report the results in the third quarter of 2014 or first quarter of 2015.

### Regulatory aspects

The medical ethics committee of the Academic Medical Center confirmed that the Medical Research Involving Human Subjects Act (WMO) does not apply to this study and that an official approval of this study by the committee is not required. The interventions are simply different means of giving valid information to physicians. The trial is supported by two grants from The Netherlands Organisation for Health Research and Development (ZonMW). The funder and the software companies involved have no role in study design, data collection and analysis, or decision to publish on this study. Furthermore, only anonymized patient data are used for analysis. Every practice has provided written consent to participate in this study via a designated GP representative.

## Discussion

This study presented an innovative CDSS and described the protocol for two RCTs to study the effects of decision support and framing on level of adherence to selected sets of ACOVE-based CRs in an outpatient setting. The system is characterized by a non interruptive presentation and real-time color-coded messages that are promptly updated based on the GP’s actions.

The CDSS is novel in the sense that it combines all of the following: it is not interruptive; chooses for a critiquing or consulting mode per rule; allows for framing advices in two styles; provides color-coded messages in real time; allows for understanding why a GP does not choose to follow a rule; and is based on CRs specifically chosen by the end users (the GPs) after Delphi-like rounds. We expect that these aspects will result in increased acceptance and thereby improve adherence to CRs. Specifically, real-time feedback has been correlated with success in decision-support implementation [15,16]. However, providing real-time feedback has traditionally been based on pop-up messages that interrupt the clinicians’ workflow and contribute to alert fatigue [17] and the perception of too many alerts is already prevalent among the clinicians in this study [18].

We considered five main levels of randomization: the level of the patient, the practitioner, the team, the center, and the CR. Below we discuss the pros and cons of each.

### At the patient level

Randomization at the patient level was considered inappropriate for a number of reasons. Most importantly, it could be confusing to the GPs to receive different alerts about different patients, and could compromise patient safety if the practitioner incorrectly interpreted the absence of an alert that he or she had seen for a previous patient to mean the absence of that problem in the current patient.

### At the level of GPs

In this scenario, which is our choice, GPs will be randomly allocated for a set of automatically verifiable CRs, to one of the two groups: 1) a group receiving CDSS feedback (intervention group) and 2) a group not receiving CDSS feedback (control group). For non automatically verifiable CRs, one group will receive the AP and the other the IN framing. The advantage of this approach is the large number of individual GPs to be randomly allocated to one of the two groups; this increases power. A disadvantage is the potential contamination through the possible exchange of information between GPs within centers and within teams. In particular, for verifiable CRs the intervention-group GPs may influence those in the control group. Similarly, there might be contamination of the control-group GPs via patients that were previously seen by the intervention-group GPs, for example, because the intervention-group GP is absent or works part-time, and the patient visit is scheduled on an irregular day for the patient.

### At the level of centers

In this scenario clusters are defined as populations of patients served by the GPs of a primary care center. GP clusters are randomly allocated to one of two groups: 1) those receiving CDSS/AP feedback and 2) those without CDSS/IN feedback (control group). Cluster-randomization avoids contamination by the effects of possible exchange of information within a cluster of GPs (center and team). However, as there are only six GP centers available to randomize for the current study, a relatively very long follow-up time would be required to compensate for the loss of power. In addition, differences between centers in terms of GPs and patients may necessitate stratification of centers during randomization, leading to further loss of power.

### At the level of CRs

In this type of design every CR is randomly assigned to GPs for decision support: some of the GPs will be in the intervention/AP group of the CR (receiving feedback or AP framing about it) and the other GPs will be in the control/IN group for that CR. This means that every GP will be in the intervention/AP group for a randomly selected subset of CRs and in the control/IN group for the complementary set of CRs. It is very probable that every GP will have a unique set of CRs for support and framing, different to any other GP. The advantage is that there are more CRs (expected to be more than 18 verifiable CRs) to be randomly allocated than centers, yielding higher power and hence a shorter minimum follow-up period required to detect an effect. The disadvantage is that contamination between GPs is still possible. However, an additional problem is that the CRs can be inter-related (for example because they pertain to the same condition, such as falls), which leads to contamination, because support for one CR may not only influence the GPʼs behavior for that CR but also for other, related CRs, even if they are not supported. The CRs must therefore be grouped together and all CRs in each group should either be supported or not. In effect, we then have a cluster-randomized trial in which the cluster consists of the CR groups. Hence the power disadvantages apply again due to the low number of resulting clusters (groups of CRs) and the possible need to stratify randomization to have similar GP and patient characteristics for each cluster.

## Trial status

Upon submission of this paper the CDSS plug-in was under development and testing. It was employed in late September 2013. Data collection, hence including patients, started from October 1st 2013, and the study will run for at least 6 months.

## Abbreviations

ACOVE: Assessing care of vulnerable elders; AP: action with positive consequences; CDSS: clinical decision support system; CR: clinical rule; CRE: clinical rule engine; DFL: dynamic floating list; EHRS: Electronic Health Records System; EMR: electronic medical record; GP: general practitioner; IN: inaction with negative consequences; LERM: logical elements rule method; QI: quality indicator; XML: eXtensible markup language.

## Competing interests

The authors declare they have no competing interests.

## Authors’ contributions

AA: conception and design of study, interpretation of data and manuscript writing. SR: conception and design, and critical revision. SE: design, interpretation of data, and manuscript writing. JW: design and critical revision. HvW: design and critical revision. MA: design and critical revision. DA: design and critical revision. SM: design, and critical revision. All authors read and approved the final manuscript.
